# Student-Driven Course-Based Undergraduate Research Experience (CUREs) Projects in Identifying Vaginal Microorganism Species Communities to Promote Scientific Literacy Skills

**DOI:** 10.3389/fpubh.2022.870301

**Published:** 2022-04-28

**Authors:** Min Wang, Su-Fang Wu, Wei-Lin Sang, Ying-Ying Zhang, Wei Liu, Ye Yang

**Affiliations:** ^1^Department of General Surgery, Shanghai General Hospital, Shanghai Jiao Tong University School of Medicine, Shanghai, China; ^2^Department of Obstetrics and Gynecology, Shanghai General Hospital, Shanghai Jiao Tong University School of Medicine, Shanghai, China; ^3^Department of Orthopedics, Shanghai General Hospital, Shanghai Jiao Tong University School of Medicine, Shanghai, China; ^4^Department of Respiratory and Critical Care Medicine, Shanghai General Hospital, Shanghai Jiao Tong University School of Medicine, Shanghai, China; ^5^Department of Educational, Shanghai General Hospital, Shanghai Jiao Tong University School of Medicine, Shanghai, China

**Keywords:** course-based undergraduate research experiences, student-driven, vaginal microorganisms, 16S rRNA gene amplicon sequencing, scientific literacy skills

## Abstract

**Objectives:**

We aim to build a students' own engagement in original microbiological course-based undergraduate research experience (CUREs) model served two research and teaching scientific purposes including students' scientific literacy skills and instructors' role, which could further be applied as contribution to broader scientific knowledge and conduct novel research in their future research experience and careers.

**Methods:**

We describe a student-driven CUREs model on the microorganism species in female vaginal using general bacterial culture techniques and high-throughput 16S rRNA gene amplicon sequencing to enable students to center experimental research method under the direction of instructors. A total of 8 undergraduate students and 5 instructors from Shanghai Jiao Tong University School of Medicine participated in the project. The CUREs were divided in four operating scopes: project planning, implementation, summarizing and feedback phases. Instructors help students to develop learning research goals.

**Results:**

This project helped students to gain “hard skills” experiences in scientific theoretical research process and technical practices. Students reached the conclusion that *Lactobacillus* species dominated the primary vaginal microbiota in reproductive-age women, 16S rRNA sequencing is a method widely applied for microbiology detection. CUREs also increased students' engagement in scientific experiments and promote 3 learning goals in “soft skills”: (1) Develop students' self-study and efficacy ability, expression capability and professional research communication skills; (2) Strengthen students' motivation and ownership in science research, overcoming failure, benefitting persistence and patience, building professional science identity, competence, and confidence in collaboration, implement spirit of rigorous and carefulness; (3) Obtain authorship, independent and logical thinking capability, summarizing ability and confidence enhancement. Instructors proposed guiding research question for the students and determine evidence in achieving pedagogical goals in CUREs.

**Conclusions:**

Our microbiological CUREs project served two scientific purposes: research and teaching, which increase students' engagement in promoting learning gains in scientific research skills, ownership, identity development, and spirit of motivation, self-efficacy, persistence, collaboration, communication, as well as opportunities to make relevant scientific discoveries. These abilities equipped them with essential foundation for the subsequent collaborative experiments and future scientific study.

## Introduction

Course-based undergraduate research experiences (CUREs) are laboratory-learning lessons to investigate an original research question driven by students' authentic participate in novel scientific study based on their own interest and curiosity early in their college careers. It is an essential teaching model characterized by students obtaining science laboratory experiences with steps of involving discovery, conducting scientific practices, including relevant research questions and promoting collaboration in the research process, which is different from other science laboratories only relied on “cookbook labs” for student (1). Unlike traditional laboratorial based courses with outcomes known to the instructor. Instead, CUREs focus on student driven inquiry and active participation in the scientific process of experiments where the outcomes are often unknown to the instructors (2). CUREs benefit both the instructors and the students. The benefits to the students include ownership of the research project through developing their own research questions and hypotheses, effective at promoting students' research self-efficacy, science identity development, and persistence in the domain (3). Students designed an original research hypothesis, summarized content knowledge from literature, implemented a research plan, worked with teams, collected and analyzed data, concluded t novel scientific findings and discussed results with their peers and faculty members (4). Current evidence in biology education indicates that CURES is effective to develop students' science identity (3) through engagement in scientific research which promote students' self-efficacy and attitudes about learning, persistence, collaboration and networking (5, 6), thus lay the foundation for future basic medical research (2, 7). While benefits to the instructor include integrating research with teaching by training students to participate in independent research ability (8). CUREs instructors' key duty is to plan research instruction, choose suitable experimental tools and methods, assess CUREs outcomes and develop students' collaborative skills so that students can successfully apply their learning to conduct real research projects (9).

Since an essential goal for successful scientific education is offering opportunities for medical students to conduct research and realize real-world scientific experiences (6). CUREs topics addressed the aspect of microbiology, cell/molecular biology and genetics, immunology, histology and biotechnology (10). In recent years, microbiology has become a focus of research and is regarded as an important part of disease diagnosis and treatment with immediate “real-world” applications. The Human Microbiome Project (HMP) recommends characterizing the abundance and diversity of microorganisms using next-generation sequencing technology (11). Bacterial metabolism is an essential part of undergraduate microbiology education (12). For females, the vaginal microorganism environment includes a diverse set of species, constitute a biological barrier to pathogenic microorganisms (13), and protect Gynecologic Cancers (14).Beneficial bacteria, such as *Lactobacillus*, account for more than 80% of all vaginal bacteria and constitute the first line of defense against invasive microorganisms. The absence of *Lactobacillus* has been associated with disturbances in the vaginal microbiota, resulting in an inflammatory vaginal disease in ~40% of healthy women and allowing infection through overgrowth of harmful anaerobes, such as *Gardnerella vaginalis* and *vulvovaginal candidiasis* (15, 16).

In this project, we employed general bacterial culture techniques and high-throughput 16S rRNA gene amplicon sequencing to explore vaginal microorganism species communities between premenopausal and postmenopausal women. Instructors were available for guidance and demonstrations of scientific technical skills and abilities. The specific focus questions guiding this exploratory study are:

1. What are the approaches used for microbiological CUREs?
2. What are the valuable skills and benefits for students by CUREs? And how did students evaluate their scientific literacy skills in the CUREs?
3. What is the instructors' role in the CUREs?
4. What is the effectiveness of CUREs education directly contributing to science ?
5. How did this CUREs project implied on other development research practices?

The purpose of our CUREs model was to engage students to participate in microbiome experimental study where they could associate theoretical knowledge to research practice through experimental repetition cycles and critical evaluation of data (17), and improve students' scientific literacy skill as critical thinking, problem solving skills and collaboration in scientific research, thus enhancing their innovation and self-authorship in scientific processes (18, 19).These essential technical abilities and spirits would equip them fitting well into other broader research-based courses and relevant to the scientific community at large, better preparing students for future careers.

## Materials and Methods

### Study Design

This study was performed in Shanghai General Hospital affiliated with Shanghai Jiao Tong University School of Medicine. A total of 8 undergraduate students, identified as S1, S2, S3, S4 on grade 3 and S5, S6, S7, S8 on grade 4 in Shanghai Jiao Tong University School of Medicine, 3 clinicians (C1, C2, C3) and 2 experimental technician (T1, T2) as instructors with backgrounds in microbiological experimental research participated in the project. They were divided into 2 groups, S1, S2, S5, S6, C1, C2, and T1 as Group 1 (G1), the remaining researchers as Group 2 (G2). Both groups participate in determining the vaginal microorganism detection method and species distribution between premenopausal and postmenopausal women under the guidance of the instructors. A total of 597 women, including 124 postmenopausal women and 473 reproductive age women, were enrolled in this study as experimental research object, among which 30 vaginal discharge samples were subjected to identification of the microbial community abundance and diversity using high-throughput 16S rRNA gene amplicon sequencing. Our project was divided into four phases termed CURE #I to #IV (Table 1), where students engaged in planning, implementing, summarizing and providing feedback, respectively. Students averagely worked on the CURE project 1–2 h daily.

**Table 1:** Stage goals, activities, and focal skills associated with each phase of the project.

**CUREs**	**Phase**	**Month**	**Activity**	**Details**
#1	Planning	1–2 January to February 2021	Reading primary literature Propose hypotheses Experimental design Online science communication with instructors	Search suitable clinical and laboratory technology to detect vaginal microorganism species community. Study culture of microorganism community techniques and high-through 16S rRNA gene amplicon sequencing to identify vaginal microorganism species between premenopausal and postmenopausal women.
#2	Implementing	3–6 March to June in 2021	Collect vaginal samples in the outpatient department	
			General bacterial culture techniques	Identify the 10 most abundant vaginal microorganism genera between the premenopausal and postmenopausal groups.
			16S rRNA gene sequencing	PCR amplification, OTU and community taxonomy system analysis at the genus level, COG-based functional structure distribution to identify species classification and analysis of colony differences at genus level.
#3	Summarizing	7–10 July to October in 2021	Data interpretation	Describe the outcome of abundance and diversity of microbial communities through 16S rRNA analysis. Compare differences in microorganism diversity in female vaginal fluids between the premenopausal and postmenopausal group.
			Statistical inference	Mann-Whitney U-tests and Benjamini-Hochberg false discovery rate correction for non-parametric data; paired *t*-test, chi-square test and one-way ANOVA for parametric data
			Graphical inference	
			Collaborative writing manuscript	
#4	Feedback	11–12 November to December in 2021	For Students and instructors	Exposing feedback loops, facing obstacles, multiple connections among outcomes.

**CURE #I**: Planning phase, From January to February 2021 (Months 1–2), students referenced papers on pubmed (https://pubmed.ncbi.nlm.nih.gov) and derived a follow-up research plan, generated common research questions about culture bacterial community techniques and 16S rRNA gene amplicon sequencing to test the vaginal microbiome in vaginal discharge. They discussed experimental design approval and project strategies with instructors using video conferencing software like Tencent Communications (https://meeting.tencent.com) or wechat, and they prepared all materials needed to conduct the research.

**CURE #II:** Implementing phase: From March to June 2021 (Months 3–6), students collected vaginal secretion specimens for bacterial culture in the outpatient department twice a week. Then, they performed microorganism detection techniques according to their lab schedule under the guidance of the instructors and recorded and organized preliminary scientific data as the basis for research.

**CURE #III:** Summarizing phase: From July to October 2021 (Months 7–10), students focused on dissemination and communication of project findings, presented a brief feasibility analysis, and verified the data similarities and differences in results and interpretation.

**Figure 1:**
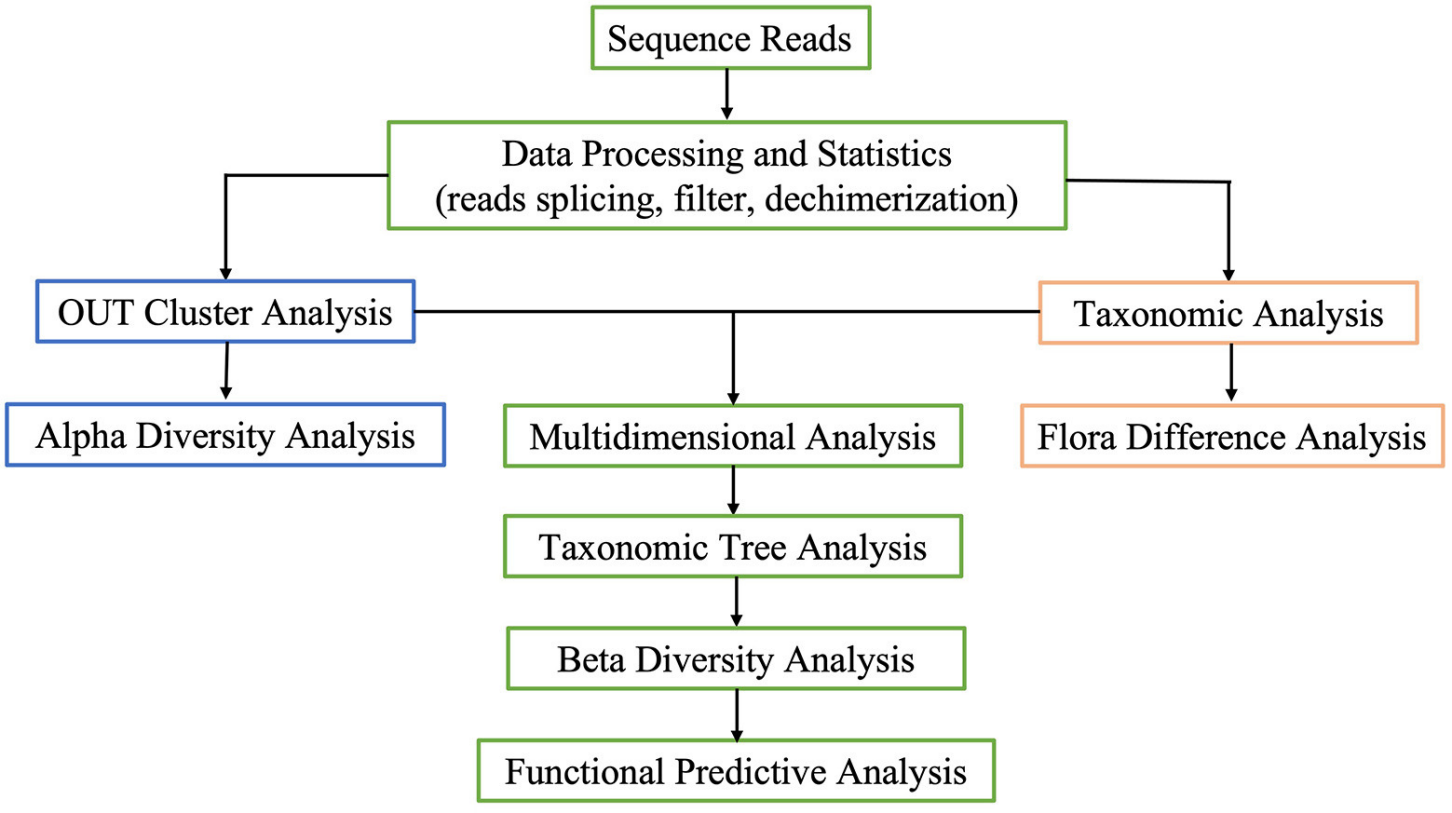
CUREs project served two research and teaching scientific purposes including students' scientific literacy skills and instructors' role.

**CURE #IV:** Feedback phase: From November to December 2021 (Months 11–12), both students and instructors conducted systematic reflection online to measure learning goals and settle problems. Recruitments and online analysis were gathered by Wen Juan Xing (https://www.wjx.cn). Each student was asked to provide their own role in facilitating the CUREs, how to resolve difficult issues, and make a short description in “hard and soft” skills with the self-evaluation score: Greatly improved: 3; Better improved: 2; General improved: 1; No improve: 0. Instructors were asked to provide assignments displayed by focus question regarded as instrument for program evaluation, analysis, ethical consideration (Figure 1).

### Vaginal Sample Collection

Students collected vaginal samples using a cotton swab under the instruction of trained gynecologists. The samples were stored at room temperature for bacterial culture or at −40°C for 5 h until DNA extraction for the 16S rRNA Amplicon Library.

### High-Throughput 16S rRNA Gene Amplicon Sequencing

NCBI's Sequence Read Archive (BioProject ID: PRJNA46877) was used as a control for vaginal microbiome 16S rDNA reference sequence analysis (20), and the generated contigs used the *Lactobacillus gasseri* (ATCC 9857) strain as the reference genome. Sequence element enrichment analysis (SEER) was used to analyze the annotated genomes for kmers of various lengths [47]. The resulting list of kmers was evaluated by likelihood-ratio p, and BLASTx and BLASTn yielded the top 50 hits. The clonal population structure of bacteria was estimated using Mash with default settings [48]. We used PCR primers that targeted the V3-V4 universal sequencing platform used 341F primers: CCCTACACGACGCTCTTCCGATCTG (barcode) CCTACGGGNGGCWGCAG and 805R primers: GACTGGAGTTCCTTGGCACCCGAGAATTCCAGACTACHVGGGTATCTAATCC. All paired-end libraries were gathered into one lane and sequenced on the Illumina MiSeq^TM^ platform at the Biomicro Center (MIT, Cambridge, MA).

### Statistical Analysis

Microorganism community distribution between premenopausal and postmenopausal women using general bacterial culture techniques were analyzed with Chi-square test. The statistical significance was set at *P* < 0.05. The students computed statistical analysis using R software version 3.2.0. The means of the flora variables were analyzed by two-tailed, paired *t*-tests (a = 0.05) using SPSS version 25.0. The filtered results for kmers used chi-square test of *P*-value <0.01. The operational taxonomic units (OTU) abundance difference between discrete categories was calculated using the Mann-Whitney U test and Benjamini-Hochberg false discovery rate correction. Hierarchical clustering and Wilcoxon tests were performed to compare genera classification. Linear discriminant analysis effect size (LEfSe), Anosim, Permutational multivariate analysis of variance (PERMANOVA) analysis and Analysis of variance (ANOVA) were carried out to compare differences in bacterial flora abundance between samples; Principal Co-ordinates Analysis (PCoA) analysis based on UniFrac was used to identify the similarities and differences in the vaginal microbial abundance matrix. Prism 8.0 was used to make the graphs.

## Result

**Figure 2:**
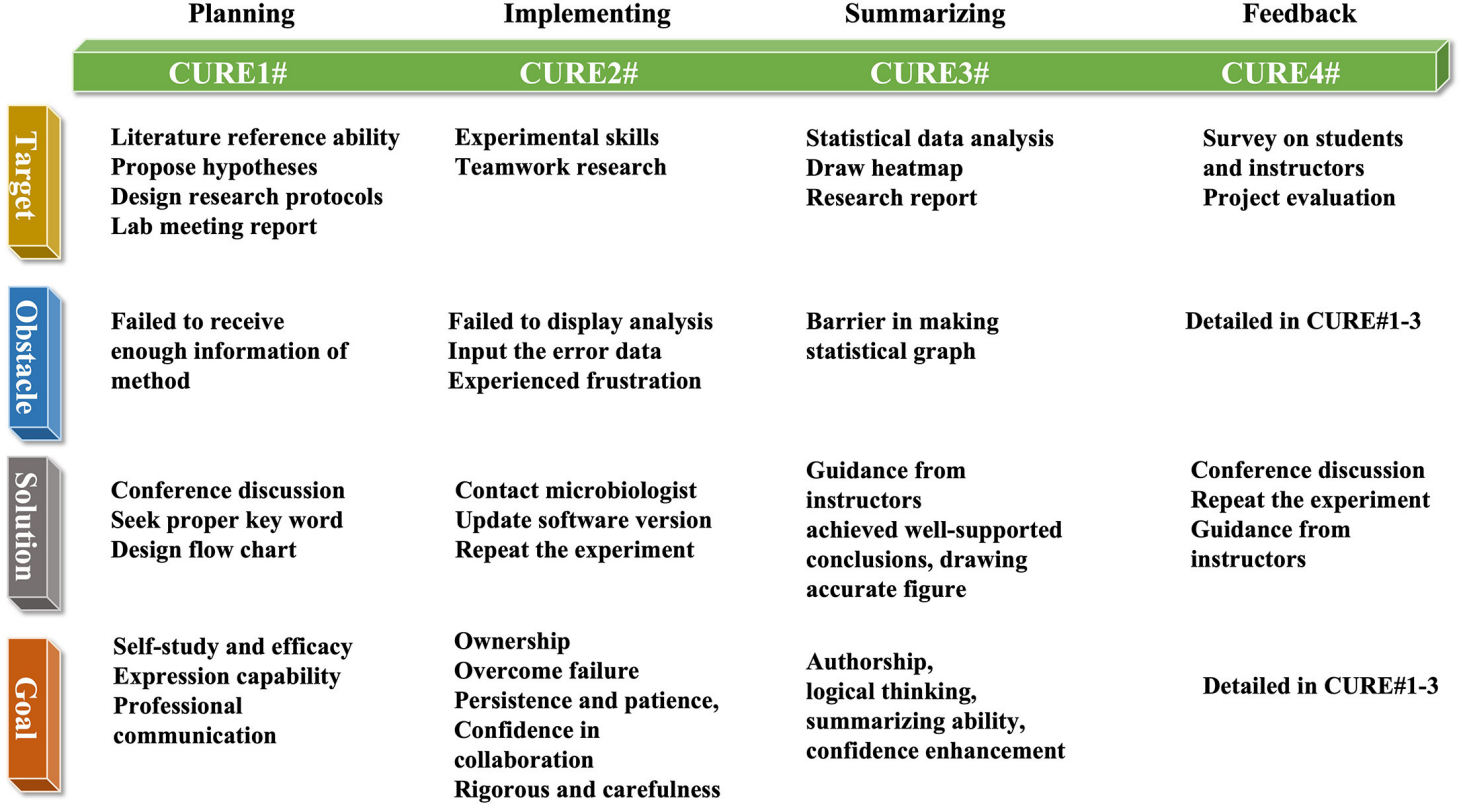
Framework in target, obstacle, solution, and goal of CUREs students reach.

CUREs project served two research and teaching scientific purposes including students' scientific literacy skills and instructors' role was displayed in Figure 1. Framework in target, obstacle, solution and goal student reach was displayed in Figure 2.

**CURE #I, Planning phase;**

**Learning Goal:**

**Hard skills (HS): Reserve foundational knowledge from primary literature, propose hypotheses, and design research protocols**.

**Soft skills (SS): Develop students' self-study and efficacy ability, expression capability, and professional research communication skills**.

During the planning phase, instructors taught each group students the skills to seek open-access literature on web-based resources “pubmed” firstly, students began to link their research to summarize the recent findings and understanding of the vaginal bacterial microbiota. At the first week, students from Group 1 faced the obstacle on seeking the reference with accurate keywords, they first referenced with key word “vaginal bacteria, vaginal lactobacillus” but failed to receive enough information on experimental method, then they made a small conference on wechat with Group 2 students and obtained new key word “vaginal microbiome, high-throughput 16S rRNA gene amplicon sequencing, et al.” from the reference they found so that they could seek more related reference (Table 2). On the next 2 weeks, the students became proficiency in the methods of retrieving literature basically, they gradually concluded vaginal microbiome plays an important role in human health and species of vaginal bacteria have been associated with reproductive disease.

**Table 2:** List of main associated literature in planning phase students prepared of Vaginal Microbiome.

**Topic:**	**Key**	**References**
The Structure and diversity of strain-level variation	Species-level diversity of vaginal microbiome communities change with respect to the reproductive stages of a woman. Vaginal microbial communities will differ in terms of number and strength of interspecies interactions, in turn have implications for the relative resistance and resilience of each community type to disturbances.	Eppinger et al. (21)
In Various Urogenital Disorders	VMB can impact the pathogenesis of urinary tract infection (UTI).	Yildirim et al. (22)
In vaginal infections and inflammatory conditions	Interplay between the cervicovaginal microbiota and the cell of immune system is determinant to prevent infections by external pathogens and to maintain an immuno-tolerant environment. Common vaginal strains as *L. gasseri* and *L. crispatus* have been used in recurrent BV.	Torcia (23)
In Gynecologic Cancers.	The vaginal microbiome, in addition to its role in common conditions such as vaginitis and HPV infection, may also have an impact on the development or prevention of gynecological cancers.	Champer et al. (24)
During pregnancy	Preterm birth is associated with increased vaginal microbiome instability compared to term birth High-risk vaginal microbiota linked to PPROM are observed closer to the time of membrane rupture, dominance of the vaginal microbiota by non-Lactobacillus spp. at any gestational age increases risk.	Stout et al. (25) Bennett et al. (26)
Vaginal Microbiome Techniques	high-throughput 16S rRNA gene amplicon sequencing.	Ricci et al. (31)

In this part, students obtained “hard skills (HS)” of literature reference methods and “soft skill (SS)” of self-study and efficacy ability, as well as became familiar with the scientific practice of developing hypotheses: Vaginal microbial communities exist in a state of dynamic equilibrium and that homeostatic mechanisms exist to provide resilience. Lactobacilli are among the most dominant populations in the vaginal system (27) and provide protection against opportunistic and pathogenic bacteria. Lactobacillus species depletion associates with the increased risk of bacterial and HPV infection (28) as well as miscarriage (29), preterm birth (30).

Next both students and instructors attended online Tencent video conferencing for communication, discussions or debates, and summarizing 2 h each week. S1, S5 from G1 and S3, S8 from G2 reported literature summary by slide presentation, S2, S6 from G1 and S4, S7 from G2 proposed their experimental hypotheses and potential outcomes about research proposals. At the beginning, some students like S1, S3, S7 lack the confidence to contact research professors to ask about questions. The instructor C1 and C2 encouraged them to draft emails or communicate online thus helped them to overcome barrier of timid and shy. During lab meeting, instructors proposed guiding research question to determine evidence in achieving pedagogical goals like: Which scientific results are we aiming for? Which skills and competencies should students be able to demonstrate? After about 2 weeks, nearly all the students felt adeptly and became more confident to discuss and form their own scientific hypotheses as well as display their research plan designing. On the other hand, students from different grades help and gradually get acquainted with each other and accumulated friendship. Both groups of students learned about how to generate high-throughput 16S rRNA gene amplicon sequencing (31) as a suitable laboratory technology to detect vaginal microorganism species community distribution theoretically. The instructors in group 2 organized group talks and summarize conference abstracts, instructors in group 1 and experimental technician T1 and T2 especially pointed out key experimental steps, such as DNA extraction, filtering OTUs, detect PCR primers targeted the V3–V4 sequencing platform and highlighting study limitations. After discussion with instructors and within groups, students carefully organized the experimental steps again and form preliminary study protocols. The instructors C2 and experimental technician provided guidance for students to ensure that the experimental strategies were well-designed and that the protocols and methods were technically feasible. In this section, students' expression capability, academic communication with team-member and instructors were improved.

**Figure 3:**
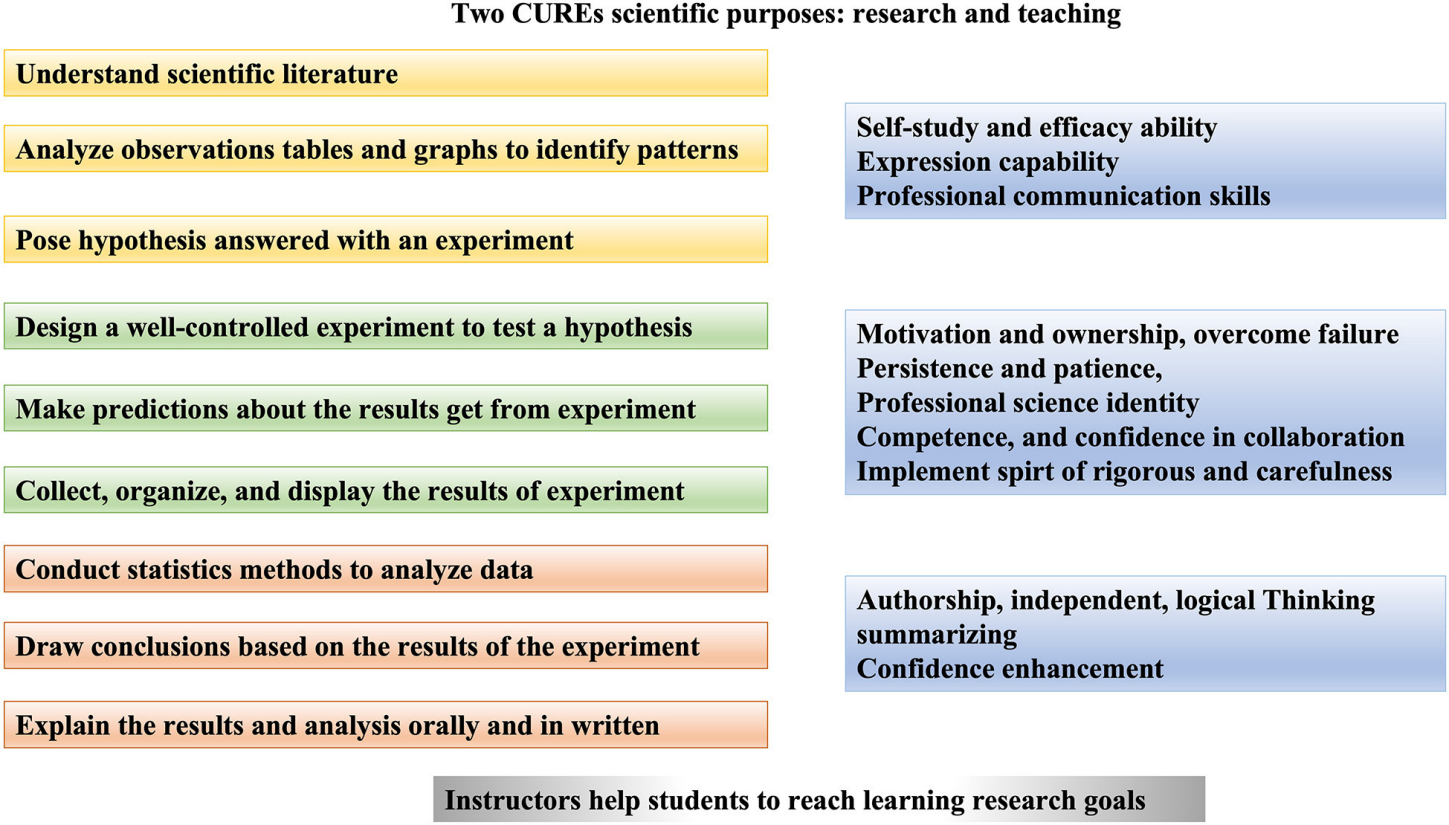
Steps of flow chart for 16S rRNA gene amplicon sequencing.

Finally, they decided to identify differential vaginal microorganism species between premenopausal and postmenopausal women using general bacterial culture techniques widely used in the clinic and 16S rRNA gene amplicon sequencing used in the laboratory. G1 and G2 students were in charge of listing step 1–5 and 6–10 of detailed experiments separately, ultimately both groups made the protocols flow chart associated with statistical analysis of 16S rRNA gene amplicon sequencing under the direction of their instructors. Instructors C1 and T2 modified flow chart order students designed (Figure 3).

### Steps of Flow Chart for 16S rRNA Gene Amplicon Sequencing

1. Sequence reads: data preprocessing and removal of chimeras and non-specific amplified sequences;
2. Data processing and statistics: reads splicing, filter and dechimerization;
3. OUT cluster analysis: cluster, distribution Venn diagram, number and cluster similarity value graph;
4. Alpha diversity analysis: Diversity index analysis, sparsity curve, Rank-abundance curve, specaccum species accumulation curve;
5. Taxonomic analysis: taxonomy, community structure composition map, single-sample multi-level species composition map, taxonomic and phylogenetic information visualization, species abundance heatmap, species abundance 3D histogram, clustering dendrogram based on species abundance samples, combined analysis of sample clustering tree and histogram, community taxonomy dendrogram;
6. Flora difference analysis: analysis of differences in bacterial flora abundance between samples, ternary plot graph, LEfSe analysis, Anosim analysis, PERMANOVA analysis, ANOVA analysis;
7. Multidimensional analysis: Principal Component (PCA) analysis, non-metric multidimensional scaling (NMDS) analysis, correlation analysis of OTU, species and environmental factors, gradient direct analysis (RDA) / correspondence analysis (CCA) analysis, network graph analysis, analysis of the relationship between microorganisms;
8. Taxonomic tree analysis: phylogenetic tree;
9. Beta diversity analysis: UniFrac analysis, multi-sample similarity tree analysis based on UniFrac, UniFrac-based heatmap, PCoA analysis based on UniFrac, UniFrac distance box plot;
10. Functional predictive analysis: PICRUSt functional analysis, functional component diagram, cluster map of samples based on functional abundance, functional abundance heatmap, combined analysis of sample clustering tree and functional histogram, function-Based PCA Analysis, feature-based NMDS Analysis, function cumulative curve, functional abundance differential analysis (Figure 3).

**CURE #II: Implementing phase;**

**Learning Goal:**

**Hard skills: Learn the foundational scientific research process and technique, conduct teamwork research under the direction of instructors**.

**Soft skills: Strengthen students' motivation and ownership in science research, overcome failure, benefit persistence and patience, build professional science identity, competence, and confidence in collaboration, implement spirit of rigorous and carefulness**.

The implementation phase was carried out over Months 3–6, and students conducted lab projects under the guide of instructors on microbiological techniques. The workflow for microbiome projects involved students collecting vaginal discharge samples in the outpatient department and detecting the vaginal microorganism community using both general bacterial culture techniques (clinical) and 16S rRNA gene sequencing (laboratory), the latter including isolation of microorganism genomic DNA from vaginal discharge samples, PCR amplification using the Illumina MiSeq platform, identification of the relative abundance of microorganism taxa using OTU tables, and distinction of the bacterial diversity across different locations using bioinformatics analyses.

### General Bacterial Culture Techniques

On the first month, students S1, S5 in G1 and S4, S8 in G2 collected vaginal discharge samples in the outpatient department to detect microorganism community distribution between premenopausal and postmenopausal women using general bacterial culture techniques by a laboratory physician and issued a report. Both group students were responsible for analyzing the results. A total of 597 women, including reproductive age women (*n* = 473, mean age 35 ± 7.07) and postmenopausal women (*n* = 124, mean age 35 ± 7.07), were enrolled in the study, among whom 41 were pregnant women and 432 were non-pregnant women. Students gathered the result that among the 20 genera identified across all samples in this study, the 10 most abundant vaginal microorganism genera between the premenopausal and postmenopausal groups were *Lactobacillus*, 56.03% vs. 77.42% (265/473 vs. 69/124); *Candida albicans* and *Streptococcus agalactiae*, 19.03% vs. 4.03% (90/473 vs. 5/124); no bacterial growth, 7.61% vs. 9.67% (36/473 vs. 12/124); *Escherichia coli* and *coagulase-negative staphylococci*, 9.73% vs. 8.87% (46/473 vs. 11/124); *Staphylococcus epidermidis*, 2.11% vs. 1.61% (10/473 vs. 2/124); *Enterococcus faecalis*, 1.90% vs. 2.42% (9/473 vs. 3/124); and other: *Proteus*, Gram-positive bacilli, *Staphylococcus aureus, hemolytic staphylococci, Klebsiella pneumoniae, Candida glabrata, Neisseria yellow, Neisseria gray, coagulase-negative bacillus, Streptococcus dysgalactiae*, and *Staphylococcus aureus*, 1.48% vs. 0.81% (7/473 vs. 1/124).

**Figure 4:**
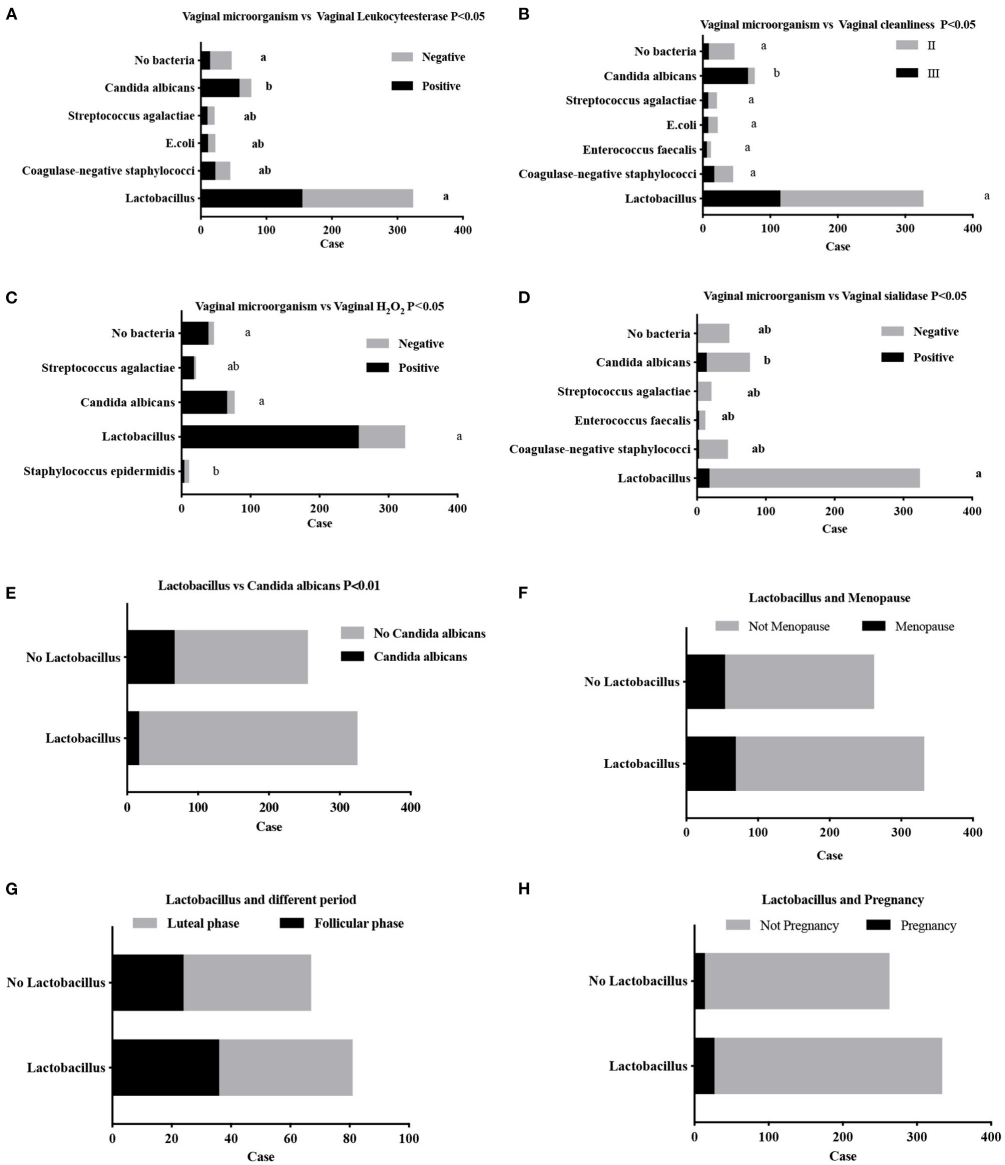
Distribution of vaginal microorganisms in premenopausal and postmenopausal women using general bacterial culture techniques. **(A,B)** Vaginal secretion triple test: *Candida albicans*, the dominant bacterial population related to a higher positive rate of leukocyte esterase **(A)** and poorer cleanliness **(B)**. *Staphylococcus epidermidis, the* main bacterial population, was related to decreased hydrogen peroxide content **(C)**, a higher sialidase content existed in the *Enterococcus faecalis* population **(D)**. Women carrying *Lactobacillus* as the primary bacterial population were less susceptible to certain pathogens, such as *Candida albicans* **(E)**. There was no significant difference in *lactobacillus* as the dominant bacteria between the premenopausal and postmenopausal groups **(F)**. There was also no significant difference in *Lactobacillus*, as the dominant bacteria, distribution in different phases of the menstrual cycle **(G)**. Among them, Lactobacillus dominated 65.9% (27/41) in pregnant women and 55.22% (307/556) of non-pregnant women **(H)**.

Combined with the vaginal secretion triple test, students found that the decrease in the proportion of *lactobacilli* led to an increase in the proportion of opportunistic infections, indicating that *lactobacilli* inhibit vaginal inflammation. *Candida albicans*, as the dominant bacterial population, was related to a higher positive rate of leukocyte esterase (*P* < 0.05) (Figure 4A) and poorer cleanliness (*P* < 0.05) (Figure 4B). *Staphylococcus epidermidis*, the main bacterial population, was related to reduced hydrogen peroxide content (*P* = 0.0039 <0.05) (Figure 4C), while higher sialidase content existed in the *Enterococcus faecalis* population (*P* = 0.0006 <0.05) (Figure 4D). Women carrying *Lactobacillus* as the main bacterial population were less susceptible to certain pathogens, such as *Candida albicans* (*P* < 0.05) (Figure 4E). There was no significant difference in *Lactobacillus*, which accounted for the dominant bacteria, between the premenopausal and postmenopausal groups (*P* = 0.9589 >0.05) (Figure 4F). There was also no significant difference in *Lactobacillus* as the dominant bacteria distributed during different menstrual cycles: the follicular phase accounted for 60% (36/60) and the luteal phase accounted for 51.10% (45/88) (*P* = 0.2875 >0.05) (Figure 4G). Among them, Lactobacillus dominated 65.9% (27/41) of the pregnant women and 55.22% (307/556) of the non-pregnant people (*P* = 0.1855 >0.05) (Figure 4H) (Supplementary Table 1). In this section, students self-mastered statistical methods of Chi-square test with two-sides and analysis the experimental data, they also learned about other statistical methods to lay the foundation for subsequent 16S experimental analysis. Instructors C1, C2, and C3 participated in verifying data.

### Identification of the Microorganism Species Community in Vaginal Discharge Using 16S rRNA Sequencing

Once students completed collecting a total of 30 vaginal discharge sample, they started to perform 16S rRNA sequencing analysis to evaluate the microbial community abundance and diversity since the second month of CURE 2#. Vaginal microbes were clustered by menopausal state, indicated as the premenopausal group, postmenopausal group, and both the premenopausal and postmenopausal groups. G1 and G2 students were in charge of conducting flow chart step 1–5 and 6–10 separately mainly under the direction of experimental technician T1, T2 and microbiologist in Shanghai Jiao Tong University School of Medicine. When G1 students encountered problems during the experiment, they discussed with students in the other group, and students in G2 assisted in reviewing literature again to find a solution and consulting experts, and vice versa. This ensures both groups of students can grasp all steps of the experiment and obtain effective allocation time.

Students in G1 used QIIME (Quantitative Insights Into Microbial Ecology, version 1.8.0) (32) to test Sequence read processing. A two-step 16S rRNA gene PCR amplicon approach was used to construct paired-end Illumina sequencing libraries (33). They followed the steps of the above flow chat to start the research under the guidance of the instructors. The raw image data files were transformed to the original sequencing sequence by Illumina MiSeq^TM^ (Phred quality score, Q_phred_) and CASAVA base calling analysis (Sequenced Reads), and chimeras and non-specific amplified sequences were removed. Genomic DNA was measured using the Qubit 2.0 DNA Assay Kit with 10 ng per sample and a final sequencing concentration of 20 pmol for PCR. They observed a total of 2,212,345 raw sequences were produced from the varieties collected from the secretion of female vaginas. After trimming, quality filtering and chimera removal, a total of 2,099,122 sequences were maintained, with an average length of 423.72 bp (Supplementary Figure 1A) (Supplementary Table 2). Although steps 1 and 2 sequence reads, data processing and statistics were relatively progressing smoothly, students realized the latter steps were more complicated than expected and failed to display OUT cluster analysis with distribution Venn diagram and process alpha diversity or taxonomic analysis at the beginning. Students in G2 referenced the literature again to seek the reason and contact the microbiologist. Instructor C3 pointed out the 16S rRNA gene PCR amplicon approach greatly rely on the software database, after the students updated the version of NCBI Blast and R package, they could successfully progress the experiment. In this section, students' motivation and ownership in science research were greatly improved. Students also realized scientific research requires many repetitions to be succeed, they should be coped frustration with persistence and patience. Instructors helped them to buffer the difficult in actual research and reduce microbiology technological barriers in experiment.

### Microbial Relationship Shared Multidimensional Analysis Based on OTU Cluster Analysis and Species Classification

To classify the community distribution, species diversity, richness of the strains and the genus sequences, it was necessary for the students to analyze the sequences of row clustering, which were divided into shared multidimensional analysis based on OTU cluster analysis species classification according to the similarity between sequences. Students in G1 tested their hypotheses that only OTUs with a relative abundance of at least 10^−6^ were maintained. Then student teams used BLAST to compare the operational taxonomic unit (OTU) sequence defined at 97% similarity and coverage to the corresponding database. The Ribosomal Database Project (RDP) was used for OTU taxonomic classification clusters of organisms with a confidence cutoff of 0.5 (34), which were grouped by DNA sequence similarity of a specific 16S rRNA taxonomic marker gene (33). RDP Naive Bayesian Classifier v.2.2 (35) filtered out the best alignment results of the OTU sequence by comparison (http://rdp.cme.msu.edu/misc/resources.jsp), while bacterial taxa with a relative abundance <1% were excluded.

Students concluded a total of 2,027,293 high-quality reads were clustered into 2,060 OTUs with 97% cutoff sequence similarity. Species with an abundance >1% or OTU information in the top 100 for bilateral testing were analyzed. Classification analysis of specific OTUs was ranked by mean decrease accuracy, and correlation analysis identified significant correlations, strong correlations, positive correlations, and negative correlations among microbial communities. Students observed differences in the bacterial communities in vaginal discharge between the premenopausal and postmenopausal groups and then selected the most abundant representative sequence under the guidance of their tutors. Both groups of students manufactured Wayne map in different colors together and considered the following diversity metrics by Mann-Whitney U test and Benjamini-Hochberg false discovery rate correction: overall counts of individual microbial OTU richness, effective counts of common and dominant OTUs, taxonomic composition, and relative abundances of classification (Supplementary Figures 1B,C) (Supplementary Table 3). Students in G1 felt their professional science identity, competence, and confidence were promoted through collaboration with team members during the experimental process.

### Composition and Differences in Vaginal Microbial Diversity

In order to identify the similarities and differences in the vaginal microbial abundance matrix revealed bacterial community diversity between premenopausal and postmenopausal groups, G2 students continue to construct PCoA based on the weighted UniFrac distance, and take the total variance of the principal components >80% as well as the covariance matrix diagonal value 1 as standard. On the routine lab meeting, G2 students reported there was no differences in the vaginal microbial abundance between the premenopausal and postmenopausal groups, which was inconsistent with literature reports. The instructors in G1 suggested to repeat step 7–9 multidimensional and beta diversity analysis again. They operated analysis again and identified student S4 input the error data. After correcting the data, G1 students concluded differences between the premenopausal and postmenopausal groups were statistically significant (Supplementary Figure 1D: eigenvalues = 1.96, var explained = 48%). The degree of similarity and relationship between the samples were imported through the distance heat map by the students and tutors (Supplementary Figure 1E). From the innermost circle to the outer circle, the composition of species was observed at the level of domain, phylum, gang, class, order, family, and genus. A single sample multilevel species composition was visually revealed from the inside out through a plurality of concentric circles at the taxonomic level (Supplementary Figure 1F) (Supplementary Table 4). Students summarized the spirit of rigorous and carefulness should be implemented throughout each procedure of the scientific research. Instructors encourage students not to get discouraged when they encounter setbacks

**CURE #III: Summarizing phase;**

**Learning Goal:**

**Hard skills: Research summaries, including data interpretation, statistical and graphical inference and collaborative writing research report**.

**Soft skills: Obtain authorship, independent and logical thinking capability, summarizing ability, confidence enhancement**.

In the summarizing phase, the students assembled their data into figures and tables, conducted bioinformatics analyses, summarized the research question, findings and results, and finally made conclusions in an original research manuscript. They need to learn SPSS and Prism as statistical and graphing software. G1 students were responsible for taxonomy and OUT analysis, while G2 students were in charge of orthologous group (COG)-based functional structure distribution and species classification. Student described their research question, proposed ideas, analyze practice data as pre-lab assignments, interpret and compare results and tried to write report papers. The instructors provided guidance, especially with thorough data analysis, achieved well-supported conclusions, drawing standard and accurate figure to enable students to facilitate the drafting process.

### Community Taxonomy System at the Genus Level

**Figure 5:**
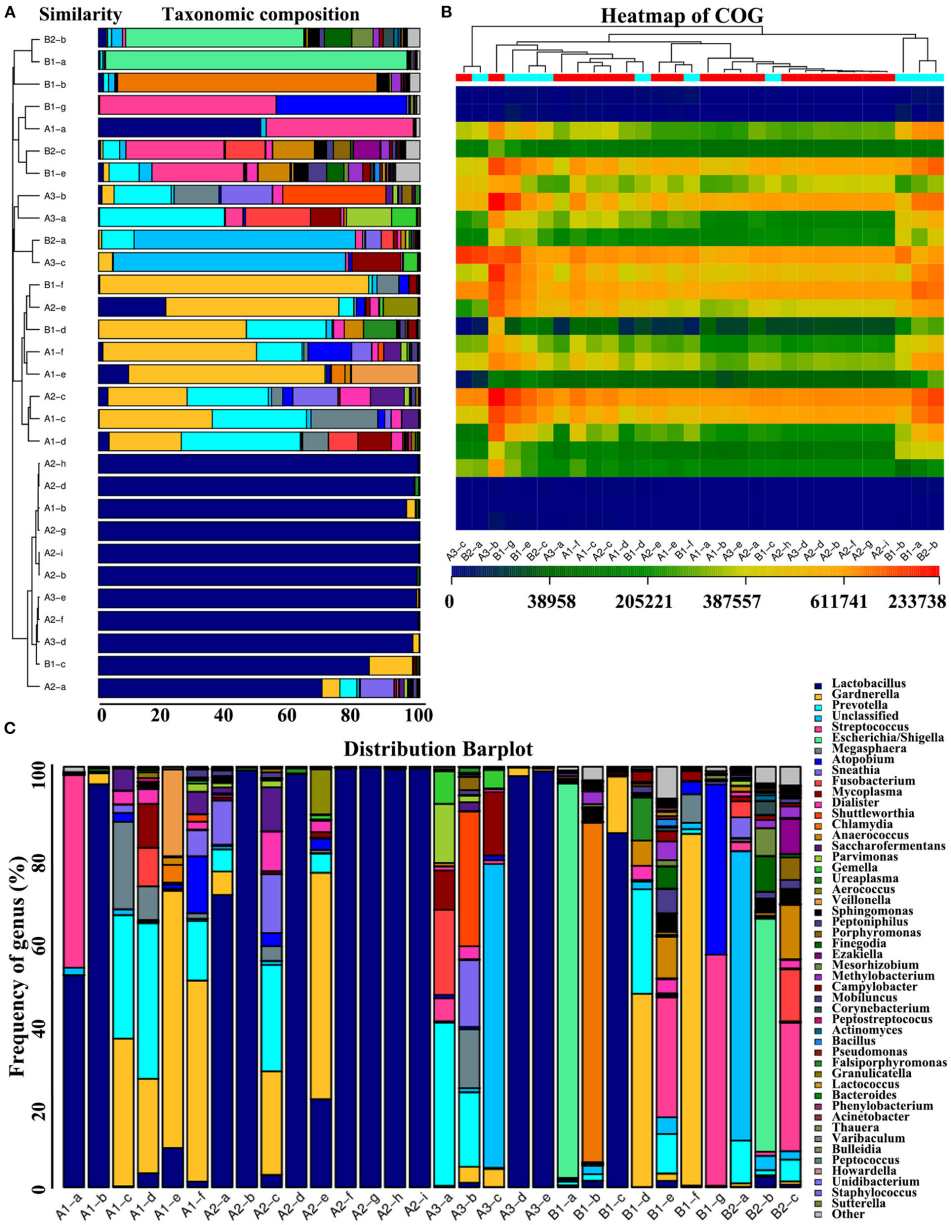
Community taxonomy system at the genus level and COG-based functional structure distribution. **(A)** Taxonomic composition distribution graph showing the relative sample microorganism classification abundance. The horizontal or vertical axis is the sample number or relative abundance ratio. The width of different colors indicates the relative abundance ratio of different species at the taxonomic level. Left panel: Bray-Curtis-based sample clustering tree diagram; middle panel part: Histogram of species abundance of clustering order; right panel: Illustration of the species. **(B)** A functional abundance heat map based on COG was drawn using the functional abundance matrix. Each column represents a sample, the row represents the function, and the color block represents the functional abundance value. The redder the color is, the higher the abundance, and the more blue the color is, and the lower the distance. Samples from the same group have the same color. Sample distance is represented by the length of the branches. The more similar the samples are, the closer the branches of the same color in the figure are from the same group. (A) RNA processing and modification; (B) Chromatin structure and dynamics; (C) Energy production and conversion; (D) Cell cycle control, cell division, chromosome partitioning; (E) Amino acid transport and metabolism; (F) Nucleotide transport and metabolism; (G) Carbohydrate transport and metabolism; (H) Coenzyme transport and metabolism; (I) Lipid transport and metabolism; (J) Translation, ribosomal structure and biogenesis; (K) Translation, ribosomal structure and biogenesis; (L) Transcription; (M) Replication, recombination and repair; (N) Cell wall/membrane/envelope biogenesis; (O) Cell motility; (P) Post-translational modification, protein turnover, and chaperones; (Q) Secondary metabolites biosynthesis, transport, and catabolism; (R) General function prediction only; (S) Function unknown; (T) Signal transduction mechanisms; (U) Intracellular trafficking, secretion, and vesicular transport; (V) Defense mechanisms; (W) Extracellular structures; (Y) Nuclear structure; (Z) Cytoskeleton; **(C)** Distribution bar plot.

Students were glad to realize that their experiment was supported by the literature, they revealed the taxonomic level for each single OTU displayed genus was calculated by the sequence probability value assigned at different levels within the V3–V4 region. V3 region amplicons of the 16S rRNA gene were analyzed on a high-throughput sequencing platform. They classified dominant species corresponding to each OTU dependent on Bergey's taxonomy using the RDP database (http://rdp.cme.msu.edu/misc/resources.jsp) (36). Similarity searches using the evolutionary relationships and abundance differences of dominant microorganisms in the sequenced samples were obtained from the entire classification system. The instructors told the students that the category name and its corresponding average abundance value were near the fulcrum, further, the microorganisms and their sequence numbers were known according to the similarity of the taxonomic composition analysis, including their relative abundance. According to the results of the taxonomy comparison of each sample, the students could visually determine the dominant flora community in the late proliferative and secretory phases of the menstrual cycle was Lactobacillus; instead of Lactobacillus, the bacteria/Shigella, Gardnerella, and Streptococcus were dominant bacteria in the postmenopausal group, analyzed by LEfSe, Anosim, PERMANOVA, and ANOVA (Figure 5A).

### Clusters of Orthologous Group (COG)-Based Functional Structure Distribution Histogram

Next, S3 and S7 students in G2 concluded differences in abundance functional prediction between the premenopausal and postmenopausal groups through the clustered heat map. They found the more similar the distance of the sample flora, the closer their position in the cluster tree above the figure. Intuitively, the functional abundance values were represented by defined shades of color, and sample function information was clustered and rearranged. They characterized differences in functional prediction between the premenopausal and postmenopausal groups included intracellular trafficking, secretion, and vesicular transport (*P* = 0.0272 <0.05); posttranslational modification, protein turnover, and chaperones (*P* = 0.0353 >0.05); RNA processing and modification (*P* = 0.0381 <0.05); inorganic ion transport and metabolism (*P* = 0.0442 <0.05); and secondary metabolite biosynthesis, transport, and catabolism (*P* = 0.0468 <0.05) (Figure 5B). The frequency of genera is displayed by a distribution bar plot graph (Figure 5C) mainly drawn by S7 with the guidance by instructors C3 (Supplementary Table 5).

### Species Classification and Analysis of Colony Differences at the Genus Level

Ultimately, S4 and S8 students imported AUC analysis to assess the accuracy of the classification models to identify differences in microorganism diversity between premenopausal and postmenopausal group varieties in female vaginal discharge. They drawn a visual circle diagram in a colored pie chart which depicted the correspondence between samples and species reflected the proportion of dominant species and the distribution of dominant species among different samples. Different colors represent different samples, and the larger the fan area of the color is, the higher the abundance of the sample on the branch. The category name and its corresponding average abundance value are near the fulcrum. Sample clustering tree and histogram combination showed the community structure of 30 samples displayed in distribution of the flora in different menstrual cycles and on postmenopausal samples.

**Figure 6:**
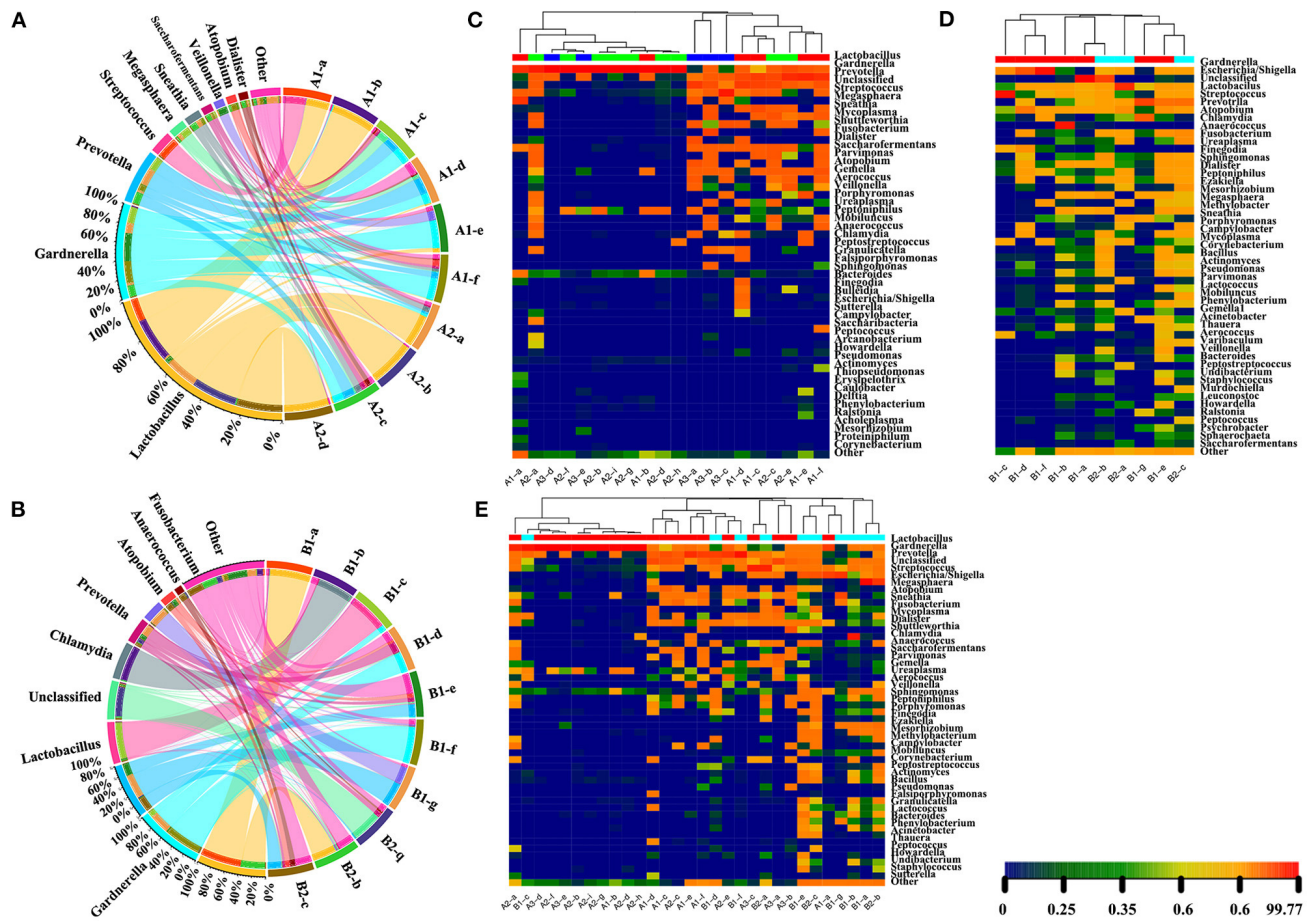
The dominant flora and heatmap of genera from the tree diagram. The dominant flora in premenopausal **(A)** and postmenopausal **(B)** groups. The difference in abundance in samples on a branch is compared using a colored pie chart. Different colors represent different samples, and the larger the fan area of the color, the higher the abundance of the sample on the branch. The semicircle on the right indicates the species abundance composition of the sample, while the left semicircle indicates the proportion of different samples in the dominant species. The length and width represent the abundance and distribution ratio of species in the sample. Heat map of the top 50 species classifications at the genus-level species abundance matrix from a tree diagram. The distribution of flora in different phases of the menstrual cycle and postmenopausal women is shown in premenopausal women **(C)**, postmenopausal **(D)** and both **(E)** groups. The same sample group has the same color. Each column in the figure represents a sample, and the row represents the community structure. The color blocks represent relative species abundance values. The redder the color, the higher the relative abundance, while the bluer the color, the lower the relative abundance. The color shade reflects the abundance of the community distribution, and clustering reflects community similarities and different distributions at each classification level.

At first, S4 and S8 students tried to draw the species abundance heat map using a species abundance matrix, however they faced barrier in adjusting proper color and chromaticity ladder, instructor C3 suggested applying contrasting colors to show statistical differences. They learned about the information that only the top 10 samples and most abundant species classifications are shown in the graph (Figures 6A,B). Thus, their made their conclusion: The dominant flora in the premenopausal group according to the menstrual cycle (the early and middle stages of proliferation, late proliferative phase and secretory phase) compared to the postmenopausal group included *Lactobacillus* (*P* = 0.0045), *Gardnerella* (*P* = 0.8088), *Prevotella* (*P* = 0.2619), *Streptococcus* (*P* = 0.0076), *Escherichia/Shigella* (*P* = 0.0757), *Megasphaera* (*P* = 0.8423), *Atopobium* (*P* = 0.8772), *Sneathia* (*P* = 0.2233), *Fusobacterium* (*P* = 0.0521), *Diallster* (*P* = 0.3904), *Saccharofermentans* (*P* = 1), *Veilbnella* (*P* = 0.2429), *Chlamydia* (*P* = 0.1566), and *Anaerococcus* (*P* = 0.0273) (Supplementary Table 6). Both the students and instructors demonstrated the dominant flora in vaginal secretions changed before and after menopause. Lactobacillus was the primary flora present before menopause. The decrease in lactobacilli postmenopause leads to opportunistic pathogens becoming the dominant bacteria. A heatmap of genera from a tree diagram clustered the samples; the more similar the sample flora, the closer the distribution of the cluster tree (Figures 6C–E). In this part, students obtained authorship, independent and logical thinking capability.

### Research Report Written by Students

Each student submitted a research summary report so that they could achieve summarizing ability and confidence enhancement. Below was a comprehensive summary report written by student S7:

Lactobacillus species including *L. crispatus, L. gasseri, L. jensenii*, and *L. iners*, dominated the primary vaginal microbiota have been associated with reproductive disease (15). 16S rRNA sequencing is a method widely applied for microbiology detection in the laboratory, while general bacterial culture is commonly implemented in clinical use (31, 37). Depending on iteration, revising and repeating experiments and troubleshooting problems to address challenges and enhance confidence, 16S rRNA sourced from a 1,500 bp gene existed in bacteria and composited with nine highly variable and species-specific regions (V1–V9) of the genome (31). Lactobacillus was the primary flora before menopause. Loss of Lactobacillus dominance promotes the colonization by anaerobic bacterial species with an increase in microbial diversity (15). The decrease in lactobacilli postmenopause leads to opportunistic pathogens, such as *Gardnerella, Prevotella, Streptococcus, Escherichia/Shigella* becoming the dominant bacteria.

Refer to 16S rRNA sequencing, comprehend OTU-based sample clustering tree diagram Wayne graph, PCoA graph, sample distance heat map representing multidimensional and species classification tree analysis. Taxonomic composition distribution graphs, distribution bar plots, COG functional abundance heat maps, and the dominant flora and heatmaps of genus form tree diagrams shown differences in microorganism diversity between premenopausal and postmenopausal women. In conclusion, maintain a stable vaginal microbiome becomes crucial for the health of a reproductive age woman (38). Lactobacillus species depletion and increased microbial diversity are characteristic of bacterial vaginosis (BV).

**CURE #IV: Feedback phase;**

**Survey detailing both the instructors' and students' self-evaluation pinions in the research, as well as project evaluation, analysis, ethical consideration**.

**Learning Goal: Benefits and effect of CUREs for both students and instructors**.

In the feedback phase, students self-reported their learning gains relative to the course elements and benefits. Students and instructors were anonymously surveyed on the effectiveness of the project and provided systematic reflection online. Recruitments were gathered by Wen Juan Xing (https://www.wjx.cn) and conducted by online analysis. Students' improvement in scientific literacy with “hard and soft” skills were evaluated with scores marked themselves covering aspect of skills in science research: literature reading, objectivity, reporting, research design, technical conduction, data statistics; as well as spirit of ownership, authorship, motivation, collaboration, which can be found in Table 3. We also adopted assignments displayed by focus question, concluded comprehensive queries with the students and instructors' answers instrument for program evaluation, analysis, ethical consideration. We hypothesized that systematic reflections by students and instructors over CUREs courses would be valuable tools to measure positive effects on student confidence, great effects and teamwork spirits in scientific research (Table 3).

**Table 3:** Evaluation by students and instructors over CUREs project.

	**Group 1**					**Group 2**				
	**Undergraduate students**				**Instructors**	**Undergraduate students**				**Instructors**
**Target**	**S1**	**S2**	**S5**	**S6**	**C1, C2, T1**	**S3**	**S4**	**S7**	**S8**	**C3, T2**
**CURE#1**										
HS	Reference review	Reference review	Reference review	Reference review	Teach reviewing skills	Reference review	Reference review	Reference review	Reference review	Teach reviewing skills
E	2	2	3	2		3	1	3	3	
SS	Self-study and efficacy									
E	2	2	3	2		3	2	3	3	
HS	Report literature summary	Proposed experimental hypotheses	Report literature summary	Proposed experimental hypotheses	Ensure experimental strategies technically feasible	Report literature summary	Proposed experimental hypotheses	Proposed experimental hypotheses	Report literature summary	Organize and summarize conference
E	3	3	2	2		2	3	2	3	
SS	Expression capability, and professional research communication skills									
E	3	3	2	3		3	3	2	3	
HS	Design Flow chart step 1–5	Design Flow chart step 1–5	Design Flow chart step 1–5	Design Flow chart step 1–5	Modify flow chart	Design Flow chart step 1–5	Design Flow chart step 6–10	Design Flow chart step 6–10	Design Flow chart step 6–10	Modify flow chart
E	2	3	2	3		3	2	3	2	
**CURE#2**										
HS	Collect sample, Analysis results	Analysis results	Collect sample, Analysis results	Analysis results	Verify data	Analysis results	Collect sample, Analysis results	Analysis results	Collect sample, Analysis results	Verify data
E	3	3	3	3		2	3	3	3	
HS	Flow chart step 1–5; Reference, find solution	Flow chart step 1–5; Reference, find solution	Flow chart step 1–5; Reference, find solution	Flow chart step 1–5; Reference, find solution	Direct experiment step by step, reduce technological barriers	Reference, find solution; Flow chart step 6–10	Reference, find solution; Flow chart step 6–10	Reference, find solution; Flow chart step 6–10	Reference, find solution; Flow chart step 6–10	Direct experiment step by step reduce technological barriers
E	2	1	2	2		2	3	2	2	
SS	Motivation and ownership									
E	3	2	3	3		3	3	2	3	
SS	Overcome failure, benefit persistence and patience									
E	3	3	3	2		3	3	3	2	
SS	Professional science identity, competence, and confidence in collaboration									
E	3	2	3	3		2	3	3	3	
SS	Spirit of rigorous and carefulness									
E	3	3	3	3		3	2	3	3	
**CURE#3**										
HS	Taxonomy and OUT analysis	Taxonomy and OUT analysis	Taxonomy and OUT analysis	Taxonomy and OUT analysis	Guidance on data analysis and statistical figure	COG functional distribution and species classification	COG functional distribution and species classification	COG functional distribution and species classification	COG functional distribution and species classification	Guidance on data analysis and statistical figure
E	2	1	2	2		1	2	2	3	
HS	Statistical analysis with SPSS and Prism	Statistical analysis with SPSS and Prism	Statistical analysis with SPSS and Prism	Statistical analysis with SPSS and Prism	Encourage drafting emails	Statistical analysis with SPSS and Prism	Statistical analysis with SPSS and Prism	Statistical analysis with SPSS and Prism	Statistical analysis with SPSS and Prism	Help them overcome barrier of shy
E	2	3	3	2		3	3	2	3	
SS	Obtain authorship, independent and logical thinking capability									
HS	Write search reports	Write search reports	Write search reports	Write search reports		Write search reports	Write search reports	Write search reports	Write search reports	
E	2	3	3	2		3	3	2	2	
SS	Achieve summarizing ability and confidence enhancement									
E	2	3	3	3		3	3	2	3	

*HS, hard skills.*

*S, soft skills.*

*E, evaluation, marked with self-evaluation score: Greatly improved: 3; Better improved: 2; General improved: 1; No improve: 0*.

**Focus question #I: What are the approaches used for microbiological CUREs?**

Our students-driven course-based undergraduate research experience (CUREs) model based on original microbiology research. In planning phase, individual students gain the ability of utilizing primary literature by using precise keywords to search related literature on pubmed, learn research methods through previous research, summarizing the theme and result of the complicated literature. The implementation phase, students conducted real lab projects under the guide of instructors on microbiological techniques. In summarizing phase students learn how to seek novel discoveries, collect, analysis and statistics data, they all grasped the operation methods of SPSS version 25.0 and Prism 8.0 and improved statistical and computational proficiency. Finally two group of students cooperated to write the scientific lab reports, which provides the capability for students to develop their ideas, obtain the content necessary to conduct their own research project and present primary data.

**Focus question #II: What are the valuable skills and benefits for students by CUREs? And how did students evaluate their scientific literacy skills in the CUREs?**

Students' improvement in scientific literacy skills were evaluated with scores marked themselves shown in Table 3. Regarding the scientific knowledge and technology students obtain. The CUREs increased students' scientific discoveries and content knowledge of microorganism species community in female vaginal fluids through understanding and generating primary scientific literature, 4 students felt greatly improved in the ability of reviewing reference and achieved self-study and efficacy spirit. Student S1, S2, S4, and S8 greatly improved their communication and collaboration skills through reporting and proposing hypotheses on lab conference with team members after the instructors help them to overcome barrier of timid and shy. Students team felt confidence in completing the team work project and this activity strengthened their understanding of the distribution of microorganism species communities. S2, S4 and S6 students especially felt their logical thinking and summarize skills have been enhanced by making protocol flow chart.

In addition, students experienced the messiness and failures of research and iterate their work through replication experiments to get success, like updating the version of database and correcting the error data. Students S1, S2, S3, S4, S5, and S6 feedback greatly progressed in overcoming failure and benefiting persistence and patience. Most of the students achieved motivation, ownership, professional science identity confidence and carefulness a lot. S1, S5, and S6 feedback that they had the opportunity to import the OUT sequences and metadata into different Figure Trees for the first time, which increased their analytical skills and enhanced science identity. S4, S7, and S8 students not only generated raw sequencing data, the weighted UniFrac distance PCoA, but also clustered OTU-based Wayne tree diagram and correlation matrix, sample distance clustering tree heat map on UniFrac, Taxonomic composition COG-based functional structure distribution graph, and the dominant genera heatmap. However, S2 and S3 students still felt different in grasping the methods of making the statistical graph. S2 and S6 felt more familiar with Chi-square test, the Mann-Whitney U test and Benjamini-Hochberg false discovery rate correction; S3 and S4 spent 1 week to grasp Hierarchical clustering and Wilcoxon tests method. S7 and S8 were mostly interested in LEfSe, Anosim, PERMANOVA, and ANOVA analysis to compare differences in bacterial flora abundance between samples. Student S7 felt greatly proud and excited since it was his first time to draw functional structure distribution heatmap successfully. Student S4 and S8 get success in making species abundance heat map after adjusting contrasting colors to show statistical differences. Most students rewarded the spirit of authorship, independent and logical thinking capability.

**Focus question #IV: What is the instructors' role in the CUREs?**

Instructors proposed guiding research question for the CUREs and determine evidence in achieving pedagogical goals. They acted as tutors to encourage student in discussion and motivated students to conduct microbiology laboratory techniques. They provided clear and comprehensive explanations when the students faced obstacles. For example, they taught students how to use PubMed to reference the primary literature, while before that, students had no experience in using reference tools on web. After students submitted the research abstract, instructors evaluate feasibility and help them to refine their experimental approach, keeping research questions a constant theme, discussing primary literature, evaluating early drafts of research questions and abstracts, and meeting with groups and ensure students would not get lost in designing their experiments. They helped student to gain skills for further education and eventual employment, provided clear and comprehensive explain. They instructed students to draw statistical figure trees with SPSS version 25.0 and Prism 8.0. Under the guidance of the instructors, students obtained positive interaction with peers and greatly felt their science identity development was improved.

## Discussion

CUREs offer research laboratory experiences to undergraduates, allowing individual students use scientific practices in a laboratory to relevant research questions, design studies, collect and analyze data on their own concern aspect (39). Multiple studies have reported the benefits of undergraduate participation in CUREs (2) including pursuing novel research questions with broad relevance and finding research opportunities (40), designing a self-directed approach to conduct experiments (18), improving scientific technical skills and persistence in science, and competitive internships, collaborating with instructors and team members (41).

Science-based CURE projects are a beneficial intervention to enable students to engage in a self-directed scientific approach and to set goals for discovery milestones (42) for research with unknown experimental outcomes (1). Currently, CURE courses cover the life science disciplines of molecular biosciences (43), biochemistry, recombinant DNA technology (44), protein expression and purification (45), molecular cell biology (46), microbiology, and genomics bioinformatics (47). Furthermore, microbiome studies are novel CURE topics integrating molecular biology (48), microbiology and phylogeny techniques (49), such as participating in answering family microbiome research questions (50), the Bean Beetle Microbiome CURE (51), students and general public volunteers mapping the gut (52), belly button (9) and oral microbiomes (53), and a CURE describing a fruit fly microbiome to assess influencing factors on the growth of Lactobacillus (54).

Our CUREs project served two scientific purposes: research and teaching. Undergraduate students were equipped with “hard skills” enabled them to create their own research proposals and hypotheses on vaginal microorganism species, conduct experiments and drive novel research, perform well relationships with team member and instructors, gain ownership of the statistical data and graph, summarize their results (55). By referencing the literature, students developed hypotheses and learned about how to discovery approaches to address questions, they seek suitable technology to detect vaginal microorganism species community between premenopausal and postmenopausal women. Then students collaborated and worked in a group to solve a problem: the dominant flora of vaginal secretions changed before and after menopause. Finally, they conducted gathering, analyzing, and interpreting data and communicating the results to solve unknown questions in the scientific community. Students also learned about different statistical methods and charts to show differences in microorganism diversity between premenopausal and postmenopausal women. Students made self-test scientific literacy skills, they realized the spirit of rigorous, carefulness, failure overcoming, authorship, persistence and confidence should be implemented throughout each procedure of the scientific research. Most of the students exhibited increased interactive behaviors, such as posing questions and one-on-one talk to promote the active and research-driven nature of the course (56). Their “soft skills” such as communication skills, project management, and teamwork spirit within a lab group were also improved.

In the CURE project, instructors directed students to read the primary scientific literature regarding vaginal microbial diversity, drive the hypothesis of implementing general bacterial culture techniques and high-throughput 16S rRNA gene amplicon sequencing to identify vaginal microorganism species between premenopausal and postmenopausal groups, facilitate strategic decisions for significant lab protocol steps, handling lab supplies and equipment safely, and managing and analyzing data throughout the process (57). They acted as facilitators (58) to inspire and guide student initiative, science identity, and participation in laboratory experience. Students achieved successful and positive interaction with peers (59) according to the feedback inquiry.

Students in our biological CURE topics started from the beginning with developing a hypothesis, present primary literature, design research, collected specimens, conduct experiment, communicate with team members and instructors, analyze data, experience the messiness and failure of research, and finally producing research. Students eventually put the theoretical knowledge learned in statistical lesion into practice through this CURE, they improved statistical and computational proficiency, this greatly strengthened their computer technology which was essential in future scientific statistics. After these series of training, students not only experienced “hard skills” of scientific theoretical knowledge and technological practices, but also gain “soft skills” covering self-study, motivation, ownership, authorship, communication, overcoming setbacks, building persistence, identity, confidence in collaboration, rigorous and carefulness, independent, logical thinking, summarizing ability in science research. Students qualified with these technical abilities and spirits would better prepare them for future research and work. Possibly we would build an opportunity to share the experiences and advantage of this type of course at a professional conference, which might be implied on other development research practices.

## Conclusions

Our microbiological CUREs issues made students to gain ownership of their research and data, which allowed them to be creative on what they were interested in, and offered them a more authentic research experience based on topics of their own interest and curiosity. Students acquired reading and scientific literature interpretation ability, designed scientific technical research, had the opportunity to do on the ground training, documenting observations, analyzing and interpreting data, and finally disseminating their research findings (60). Apart from the above promising advantages, obstacles still exist for CURE implementation with limited access to complex research methods for individual undergraduate students to conduct experiments. Since high-throughput 16S rRNA gene amplicon sequencing involved difficult steps including microorganism genomic DNA isolation, PCR primers design and amplification, OUT abundance identification, etc., which required technical staff with professional background. Students followed the complex produce with technicians together to operate certain experiments. Also, we conducted microorganism CUREs teaching project in a limited cohort of 8 students as a pilot. Further, it might be extended to a larger number of students in other development teaching course practices. We hope that experiences from our microorganism CUREs project may be helpful for other researchers to design educational CUREs projects.

## Data Availability Statement

The data presented in the study are deposited in the BioSample database repository, accession numberSUB11147188, BioProject ID: PRJNA826073. The project information will be accessible with the following link (http://www.ncbi.nlm.nih.gov/bioproject/826073), and submission (SUB11147188) has been released now on 2022- 04-15. BioSample accessions: SAMN27547963, SAMN27547964, SAMN27547965, SAMN27547966, SAMN27547967, SAMN27547968, Temporary SubmissionID: SUB11147188.

## Ethics Statement

The studies involving human participants were reviewed and approved by Shanghai General Hospital Institutional Review Board. The patients/participants provided their written informed consent to participate in this study.

## Author Contributions

YY and MW analyzed and interpreted the data regarding the CURE program. MW collected information and participated in teaching. YY, S-FW, WL, Y-YZ, and W-LS worked equally as major contributor in writing the manuscript. All authors read and approved the final manuscript.

## Funding

This work was supported by Virtual simulation experiment teaching course of Shanghai Jiao Tong University School of Medicine (2021), Postgraduate Education Research Project of Nanjing Medical University (2021), National Natural Science Foundation of China (81902628), Clinical Research Project of Shanghai Health Commission (202040455), and Songjiang District Science and Technology Research (Medical and Health) Project, Shanghai.

## Conflict of Interest

The authors declare that the research was conducted in the absence of any commercial or financial relationships that could be construed as a potential conflict of interest.

## Publisher's Note

All claims expressed in this article are solely those of the authors and do not necessarily represent those of their affiliated organizations, or those of the publisher, the editors and the reviewers. Any product that may be evaluated in this article, or claim that may be made by its manufacturer, is not guaranteed or endorsed by the publisher.

## Supplementary Material

Supplementary Figure 1 Multidimensional and species classification tree analysis using 16S rRNA sequencing in premenopausal women, postmenopausal women, and both groups. **(A)** Raw sequencing data. **(B)** OTU-based sample clustering tree diagram by Wayne graph. Different samples (**A**- premenopausal group, **B**- postmenopausal group) are represented by different colors for specific or common OTUs. The ellipse size represents the absolute value of the correlation coefficient. The correlation of left or right oblique represents a positive or negative correlation, and the color changes with the right color scale. Only results with *p*-values <0.05 are shown in the figure. Correlations of OTU abundance with group, calculated using a two-sided *t*-test, are indicated as positive (blue, t statistic > 0 and *p*-value <0.05), negative (red, t statistic <0 and *p*-value <0.05), and no correlation (gray, *p*-value > 0.05). **(C)** OTU-based correlation matrix. The corrplot package was used to draw the correlation matrix. The left or right oblique is positively or negatively correlated, and the color varies with the right gradation. Only the results with *p*-values <0.05 are shown in the figure. **(D)** PCoA represents the weighted UniFrac distance. **(E)** Sample distance heat map of the clustering tree based on UniFrac. The distance value is represented by the color block. The redder the color is, the closer the distance and the higher the similarity between the samples. The bluer the distance is, the further the distance. Error plot for comparison of differences. The left panel shows the abundance ratio of the different species classifications in the two groups, with the difference between the species abundance in the 95% confidence interval, and the right panel shows the *p*-value, *P* < 0.05. **(F)** Single sample multilevel species composition map. Circle size represents abundance. The outer ring is a heat map, and each ring represents a sample (group). Each sample corresponds to one color, and the depth of color changes with the abundance of the species.

Supplementary Table 1 Abundant vaginal microorganism genera between the premenopausal and postmenopausal group.

Supplementary Table 2 Vaginal microorganism raw sequences.

Supplementary Table 3 Microbial relationship shared multi-dimensional analysis based on OTU cluster analysis and species classification.

Supplementary Table 4 Composition and Differences of Vaginal Microbial Diversity.

Supplementary Table 5 Clusters of Orthologous Groups (COG)-based functional structure distribution histogram.

Supplementary Table 6 Species classification and analysis of colony differences at genus level.

## Abbreviations

ANOVA: Analysis of variance
CCA: correspondence analysis
COG: clusters of Orthologous Groups
CURES: course-based undergraduate research experiences
LEfSe: Linear discriminant analysis effect size
NMDS: non-metric multidimensional scaling
OTUs: operational taxonomic units
PCA: Principal Component
PCoA: Principal Co-ordinates Analysis
PERMANOVA: Permutational multivariate analysis of variance
RDA: gradient direct analysis
RDP: Ribosomal Database Project
SEER: sequence element enrichment analysis.
