# Corrigendum: Student-driven course-based undergraduate research experience (CUREs) projects in identifying vaginal microorganism species communities to promote scientific literacy skills

**DOI:** 10.3389/fpubh.2025.1576281

**Published:** 2025-04-04

**Authors:** Min Wang, Su-Fang Wu, Wei-Lin Sang, Ying-Ying Zhang, Wei Liu, Ye Yang

**Affiliations:** ^1^Department of General Surgery, Shanghai General Hospital, Shanghai Jiao Tong University School of Medicine, Shanghai, China; ^2^Department of Obstetrics and Gynecology, Shanghai General Hospital, Shanghai Jiao Tong University School of Medicine, Shanghai, China; ^3^Department of Orthopedics, Shanghai General Hospital, Shanghai Jiao Tong University School of Medicine, Shanghai, China; ^4^Department of Respiratory and Critical Care Medicine, Shanghai General Hospital, Shanghai Jiao Tong University School of Medicine, Shanghai, China; ^5^Department of Educational, Shanghai General Hospital, Shanghai Jiao Tong University School of Medicine, Shanghai, China

**Keywords:** course-based undergraduate research experiences, student-driven, vaginal microorganisms, 16S rRNA gene amplicon sequencing, scientific literacy skills

In the published article, there was an error in the author list order. It was originally “Ye Yang^1†^, Min Wang^2†^, Wei-Lin Sang^3*^, Ying-Ying Zhang^4*^, Wei Liu^5*^ and Su-Fang Wu^1*^” The corrected author list appears below.

Min Wang^1†^, Su-Fang Wu^2†^, Wei-Lin Sang^3*^, Ying-Ying Zhang^4*^, Wei Liu^5*^and Ye Yang^2*^

In the published article, there was an error in the correspondence list. It was originally “Wei Liu, wei.liu@shgh.cn; Su-Fang Wu, wusufang73@163.com; Ying-Ying Zhang, alphafly@163.com; Wei-Lin Sang, weilin.sang@shgh.cn”. The corrected correspondence list appears below.

Ye Yang, ye.yang@shgh.cn

Wei Liu, wei.liu@shgh.cn

Ying-Ying Zhang, alphafly@163.com

Wei-Lin Sang, weilin.sang@shgh.cn

In the published article, there was an error in affiliation(s) [1,2]. Instead of

“^1^Department of Obstetrics and Gynecology, Shanghai General Hospital, Shanghai Jiao Tong University School of Medicine, Shanghai, China, ^2^Department of General Surgery, Shanghai General Hospital, Shanghai Jiao Tong University School of Medicine, Shanghai, China”, it should be

“^1^Department of General Surgery, Shanghai General Hospital, Shanghai Jiao Tong University School of Medicine, Shanghai, China, ^2^Department of Obstetrics and Gynecology, Shanghai General Hospital, Shanghai Jiao Tong University School of Medicine, Shanghai, China”.

The authors apologize for this error and state that this does not change the scientific conclusions of the article in any way. The original article has been updated.

