# Supplementary figures and images for: Student-Driven Course-Based Undergraduate Research Experience (CUREs) Projects in Identifying Vaginal Microorganism Species Communities to Promote Scientific Literacy Skills
Source: Front Public Health. 2022 Apr 28;10:870301. doi: 10.3389/fpubh.2022.870301 (PMC9096218; doi:10.3389/fpubh.2022.870301)

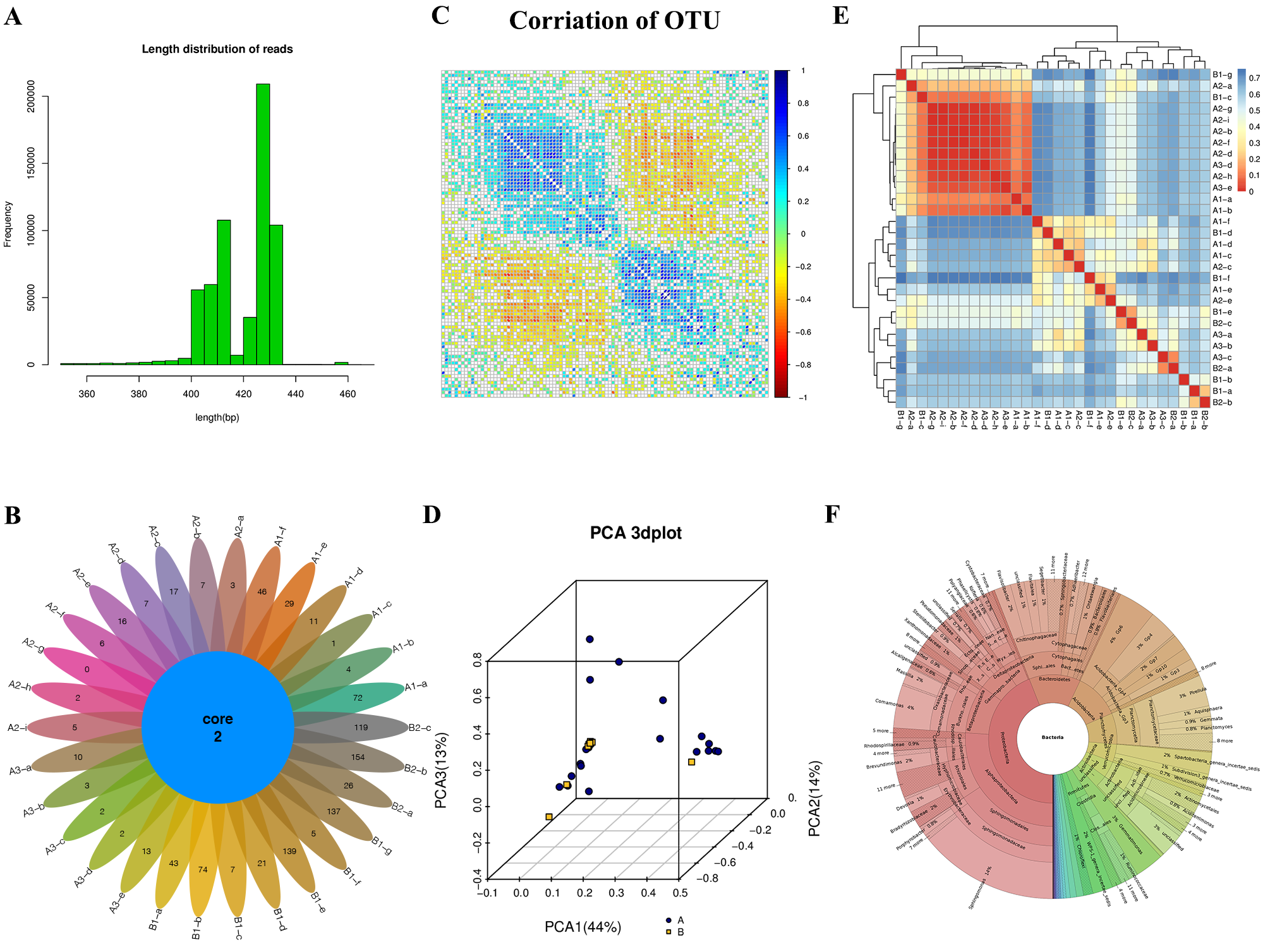

Supplement: Supplementary Figure 1 — Multidimensional and species classification tree analysis using 16S rRNA sequencing in premenopausal women, postmenopausal women, and both groups. (A) Raw sequencing data. (B) OTU-based sample clustering tree diagram by Wayne graph. Different samples (A- premenopausal group, B- postmenopausal group) are represented by different colors for specific or common OTUs. The ellipse size represents the absolute value of the correlation coefficient. The correlation of left or right oblique represents a positive or negative correlation, and the color changes with the right color scale. Only results with p-values <0.05 are shown in the figure. Correlations of OTU abundance with group, calculated using a two-sided t-test, are indicated as positive (blue, t statistic > 0 and p-value <0.05), negative (red, t statistic <0 and p-value <0.05), and no correlation (gray, p-value > 0.05). (C) OTU-based correlation matrix. The corrplot package was used to draw the correlation matrix. The left or right oblique is positively or negatively correlated, and the color varies with the right gradation. Only the results with p-values <0.05 are shown in the figure. (D) PCoA represents the weighted UniFrac distance. (E) Sample distance heat map of the clustering tree based on UniFrac. The distance value is represented by the color block. The redder the color is, the closer the distance and the higher the similarity between the samples. The bluer the distance is, the further the distance. Error plot for comparison of differences. The left panel shows the abundance ratio of the different species classifications in the two groups, with the difference between the species abundance in the 95% confidence interval, and the right panel shows the p-value, P < 0.05. (F) Single sample multilevel species composition map. Circle size represents abundance. The outer ring is a heat map, and each ring represents a sample (group). Each sample corresponds to one color, and the depth of color changes with the abunda [file Image_1.tif]
